# Mitochondrial Dysfunction: Different Routes to Alzheimer's Disease Therapy

**DOI:** 10.1155/2014/780179

**Published:** 2014-08-20

**Authors:** Pasquale Picone, Domenico Nuzzo, Luca Caruana, Valeria Scafidi, Marta Di Carlo

**Affiliations:** Istituto di Biomedicina ed Immunologia Molecolare (IBIM) “Alberto Monroy,” CNR, via Ugo La Malfa 153, 90146 Palermo, Italy

## Abstract

Mitochondria are dynamic ATP-generating organelle which contribute to many cellular functions including bioenergetics processes, intracellular calcium regulation, alteration of reduction-oxidation potential of cells, free radical scavenging, and activation of caspase mediated cell death. Mitochondrial functions can be negatively affected by amyloid *β* peptide (A*β*), an important component in Alzheimer's disease (AD) pathogenesis, and A*β* can interact with mitochondria and cause mitochondrial dysfunction. One of the most accepted hypotheses for AD onset implicates that mitochondrial dysfunction and oxidative stress are one of the primary events in the insurgence of the pathology. Here, we examine structural and functional mitochondrial changes in presence of A*β*. In particular we review data concerning A*β* import into mitochondrion and its involvement in mitochondrial oxidative stress, bioenergetics, biogenesis, trafficking, mitochondrial permeability transition pore (mPTP) formation, and mitochondrial protein interaction. Moreover, the development of AD therapy targeting mitochondria is also discussed.

## 1. Introduction

Alzheimer's disease (AD) is an age-related progressive neurodegenerative disorder characterized by impairment of cognitive function. The neuropathology of AD concerns two neurodegenerative processes: amyloidogenesis, leading to the presence of extracellular amyloid *β*-peptide (A*β*) deposition, and neurofibrillary degeneration, corresponding to the formation of intracellular tangles composed of phosphorylated Tau protein [1]. The presence of these abnormal structures leads to neuronal dysfunction and cell death. Many lines of evidence suggest that the mitochondrion plays a central role in neurodegenerative diseases, including AD [2], and the so called mitochondrial cascade hypothesis proposes that mitochondrial dysfunction is the primary event in AD pathology [3]. Mitochondrion is a cellular organelle required for bioenergetics processes and is also involved in amino-acid, lipid, and steroid metabolism, calcium homeostasis, free radicals production, and apoptosis triggering. In the brain, where there is the highest energy request and consumption, the number of mitochondria is elevated mainly in the synapses and impairment could be a serious threat for neurons survival.

Many aspects of the strict link between mitochondrial dysfunction and AD remain to be elucidated, but some evidence indicates that the progressive accumulation of A*β* in mitochondria could be the relationship for mitochondria-mediated toxicity [4]. The Amyloid Precursor Protein (APP) was, indeed, found accumulated in the mitochondrial import channels and A*β* was found interacting with some mitochondrial proteins [5].

Here we dissect different points in which the mitochondrial functionality could be affected by A*β* presence, producing evidence from studies on AD human postmortem brains as well as cellular and AD animal models. Moreover, we analyze how these vulnerable points for A*β*-mediated mitochondrial dysfunction may be distinctive and perhaps complementary therapeutic intervention targets for AD.

## 2. Mitochondria in Neuronal Cells 

High metabolic energy is required by human brain for its function. Since neurons have a limited glycolytic capacity this kind of cells is extremely dependent on mitochondrial energy production [6]. Neurons are cells with a particular morphology extending their axons and dendrites from millimeters up to a meter. Mitochondria are spread along the cells probably with a major presence in some regions of the neurons, as the synapses, that have the highest demand for ATP production and energy consumption. When a transmitter is released by a synapsis several ion channels are opened in the postsynaptic membrane to permit influx of ions. This pumping needs high energy consumption. Moreover, ATP produced by mitochondria is also consumed, together with all the normal requirements of a cell, for action potential to restore ion gradients and axonal transport. An imaging study established that a quiescent cortical neuron expends 4.7 billion ATP molecules per second [7]. A correct distribution of mitochondria into the neuronal regions in which a local demand for ATP or Ca^2+^ is necessary results to be important. Thus, trafficking of mitochondria is essential for neurons survival. Biochemical and imaging studies have demonstrated that mitochondrial translocation involves a motor/adaptor complex formed by microtubule-based motors proteins, as kinesin and dynein, together with two mitochondrion-specific proteins, milton and miro [8]. Moreover, mitochondrion together with energy metabolism discussed above plays in neurons a pivotal role in cell survival and death by regulating apoptotic pathways and contributing to different cellular functions including intracellular calcium homeostasis, maintaining the cellular redox potential, cell cycle regulation, and synaptic plasticity [9]. Mitochondrion has a role in establishing the polarity by reducing the Ca^2+^ concentration at the base of the presumptive axon, especially in neurons, thereby promoting polymerization of microtubules and the rapid neuronal growth and differentiation, accompanied by an increased amount of the mitochondria number per cell. This observation was achieved by studies in which treatment with chloramphenicol, an inhibitor of mitochondrial protein synthesis, prevents differentiation of the cells whereas oligomycin, an inhibitor of the mitochondrial ATP synthase, does not, thus suggesting that increased mitochondrial mass, but not ATP production, is required for neuronal differentiation [10]. Mitochondrial Ca^2+^ affects neurogenesis as suggested by results showing that an increase of mitochondrial fusion and intramitochondrial Ca^2+^ levels is observable when neuroblastoma cells are induced to stop division and begin differentiation in neuro-like cells [11]. During axons and dendrites differentiation, their growth and synaptic junction may be influenced by mitochondrial motility and functions. During the formation of axonal branches mitochondria respond to changes by modifying their entry into branches; the process does not require an active growth cone, suggesting the involvement of a different mechanism [12].

Furthermore, another intriguing mitochondrial role occurs during the self-renewal of neural stem cells. Studies on mouse embryonic stem (ES) cells suggest that the proliferative capacity is correlated with low mitochondrial oxygen consumption and high levels of glycolytic activity [13]. Human ES cells exhibit an anaerobic metabolic profile. When somatic cells are induced to revert to an ES cell-like phenotype their mitochondria follow the same fate changing their morphology, subcellular distribution, biogenesis, and ROS and ATP production [14].

On the basis of these observations it is easy to understand how several inherited diseases are caused by mutations in mitochondrial DNA, and cells as muscle cells and neurons, with high energy demands, are the most affected of these disorders. Furthermore, impaired mitochondrial activity causes the most common neurodegenerative disorders, such as Alzheimer's, Parkinson's and Huntington's diseases, stroke, and psychiatric disorders.

## 3. APP Metabolism and A**β** Generation

**Figure 1:**
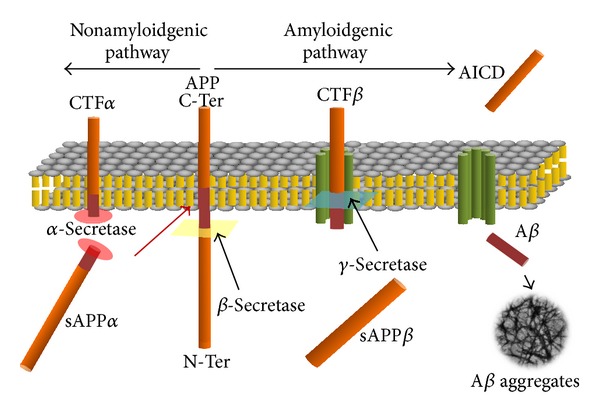
Nonamyloidgenic or amyloidgenic pathways are originated by different APP processing: the combined cleavage of *α*- and *γ*-secretase produces the sAPP*α* and CTF-*α* fragments preventing A*β* generation; in contrast, *β*-secretase cleaves in a different site of APP thus originating, together with *γ*-secretase complex, the sAPP*β* and A*β* fragments and the intracellular AICD fragment. A*β* through a misfolding step forms fibrillar aggregates.

Alzheimer's disease is a devastating neurodegenerative disorder with a progressive cognitive impairment and dementia. AD pathogenesis is believed to be triggered by the A*β* accumulation, which is due to overproduction of A*β* and/or the failure of the clearance mechanisms. A*β* is generated by sequential cleavages of its larger precursor, a protein called amyloid precursor protein (APP). APP is an integral membrane protein with a single membrane spanning domain, a large extracellular glycosylated N terminus and a shorter cytoplasmic C terminus. APP is produced in several different isoforms and the most abundant form in brain (APP695) is produced mainly by neurons and diverges from the longest one because of the lacking of a kunitz-type protease inhibitor sequence in its ectodomain [15, 16]. APP processing is divided into the nonamyloidogenic pathway and the amyloidogenic pathway (see Figure 1). The nonamyloidgenic processing is initiated by *α*-secretases, cleaving within the A*β* domain and preventing the release of A*β* [17]. Alternatively, *β*-secretase (BACE1) cleavage initiates the amyloidogenic pathway and generates the soluble *β*-secreted APP (sAPP*β*) and the membrane bound fragment CTF*β*. Both fragments are substrates for *γ*-secretase, a multisubunit protease complex comprising proteins as presenilin 1 or 2 (PS1, PS2) [18–21]. The further processing of CTF*β* releases the amyloidogenic A*β* fragment [22, 23]. Interestingly, *γ*-secretase seems to cleave the CTF*β* exactly in the middle of the membrane domain [24], suggesting the hypothesis that the formation of different A*β* species (A*β*_38_, A*β*_40_, and A*β*_42_) is dependent on the membrane properties. Each A*β* species have different lipophilic properties and different tendencies to form A*β* oligomers and aggregates. Notably, the ratio A*β*_40_/A*β*_42_ is of clinical relevance in AD [25]. However, in AD, the produced A*β* misfolds and self-aggregates into oligomers of various sizes and forms, up to produce diffuse amyloid neuritic plaques. A*β* oligomers and plaques are potent synaptotoxins, block proteasome function, inhibit mitochondrial activity, alter intracellular Ca^2+^ levels, and stimulate inflammatory processes.

## 4. Mitochondrial A**β**

A*β*, as well as its extracellular canonical localization, has been found in different subcellular compartment including the endoplasmic reticulum (ER), the Golgi apparatus or the* trans*-Golgi network, the early, late, or recycling endosomes, and the lysosome, where probably is generated [26]. However, presence of A*β* has been also observed in mitochondria [27] and studies from different independent groups have clearly demonstrated that A*β* progressively accumulates within mitochondria of both human AD brain and Tg mouse models for AD [28]. Moreover, finding indicates that accumulation of A*β* in mitochondria begins before the occurrence of the extracellular deposition as demonstrated by experiments in which its presence in Tg mA*β*PP mice arises as early as 4-5 months and increases with aging [29]. The presence of A*β* in mitochondria was also evidenced by an interesting study in which APP modulates cell death through interaction with a newly identified mitochondrial membrane proapoptotic protein, called Appoptosin, involved in the heme synthesis [30]. These findings raise the question whether A*β* in mitochondria is* in situ* generated or imported. Several observations from different groups and experimental approaches demonstrated that A*β* is not locally produced. This hypothesis is supported by the fact that the ability of *γ*-secretase to cleave APP associated with mitochondria is not known [31, 32]. Thus, it is supposed that A*β* is derived from its extracellular or intracellular pool and that a cellular trafficking is involved in the internalization of A*β* in mitochondria. Recent findings, using isolated rat mitochondria, have shown that a specific uptake mechanism for import of A*β* in mitochondria involves the translocase of the outer membrane (TOM) complex [33]. This data was supported by the fact that extracellularly applied A*β* is internalized in cells and colocalizes with mitochondrial markers and that is associated with the mitochondrial inner membrane after import. Other evidences come from experiments using confocal microscopy showing that the A*β*_42_ fragments colocalize with complex II of the respiratory chain, the outer cell membrane, the mitochondrial membrane, and the mitochondrial chaperon matrix protein Hsp60 [34, 35].

## 5. Mitochondrial Dysfunction and Oxidative Stress Induced By A**β**

Mitochondria have a pivotal role in cellular energy metabolism but are also involved in amino-acid, lipid, and steroid metabolism, modulation of cellular calcium levels, production of free radicals, and regulation of apoptosis, key features of neurodegeneration. Despite extensive research efforts to understand the pathophysiology of neurological diseases with respect to mitochondrial dysfunction, the exact mechanism is still not established. However, it seems clear that mitochondrial dysfunction plays a well-defined role in neurodegenerative diseases, making it a topic of high interest in neuroscience research today. An immediate consequence of mitochondrial dysfunction is the increase of reactive oxygen species (ROS) production that promotes oxidative damage to DNA, RNA, proteins, and lipids. Mitochondria are the primary cellular consumers of oxygen and contain numerous redox enzymes capable of transferring single electrons to oxygen, generating the superoxide (O_2_^−^) molecules. Mitochondria also contain an extensive antioxidant defense system to detoxify the generated ROS. When mitochondria are damaged, the antioxidant defense decreases thus increasing ROS production that can further damage mitochondria, causing more free radicals generation and loss or depletion of antioxidant capacity. Under normal physiological conditions, ROS act as “redox messengers” regulating intracellular signalling, whereas imbalance of ROS induces irreversible damage to cellular components leading to cell death. Mitochondrial ROS can originate from multiple reactions in the TCA cycle and/or in the respiratory chain. Several* in vitro *studies suggested a link between elevated A*β* levels, mitochondrial dysfunction, and oxidative stress, all factors that contribute to AD pathogenesis. Moreover, we reported that A*β* is a key factor in free radical generation, oxidative damage, and mitochondrial dysfunction, activating a cascade of events leading to neurodegeneration in neuroblastoma LAN5 cells [36–38] and sea urchin model system [39].

Mitochondrial A*β* presence strongly influences mitochondrial respiratory function, ROS production rates, and alters mitochondrial membrane potential in different brain regions of AD mouse models. Moreover, a different mitochondrial distribution was evidenced. The elevated A*β* presence and the mitochondrial dysfunction were found in hippocampus and cortex, brain regions devoted to memory, whereas lower levels were found in striatum and amygdala. A striking association between mitochondrial impairment and cognitive dysfunction in the A*β*PP and A*β*PP/PS1 mice was also found. This is the first demonstration of an association between mitochondrial A*β* levels, mitochondrial dysfunction, and cognitive impairment in AD transgenic mice [40]. Xie et al. for the first time showed how the structure and function of mitochondria changed in the living brain of transgenic animals developing amyloid deposits, by using intravital multiphoton imaging, with a range of fluorescent markers. The authors observed that severe impairments were limited in the brain regions close to the A*β* plaques. In these regions a decreased number of mitochondria were found; some of them were dystrophic and fragmented and the survived ones showed a reduced membrane potential. Both neuronal soma and neuritis with oxidative stress show severe alterations in mitochondrial membrane potential. These results provide* in vivo* evidence that A*β* plaques can be focal sources of toxicity leading to severe structural and functional abnormalities in mitochondria [41]. Oxidative stress may activate signalling pathways that alter APP or Tau processing. For example, oxidative stress increases the expression of *β*-secretase through activation of c-Jun amino-terminal kinase and p38 mitogen-activated protein kinase (MAPK) [42] and increases aberrant Tau phosphorylation by activation of glycogen synthase kinase 3-*β* (GSK3-*β*) [43]. Oxidant-induced inactivation of specific molecules may also be important. Using a proteomic approach, the prolyl isomerase PIN1 was found to be particularly sensitive to oxidative damage, being highly downregulated and oxidized in hippocampus of AD patients [44]. The oxidative modification of PIN1 was related to the loss of isomerase activity that is retained critical for neurofibrillary tangle formation. Later studies have demonstrated that PIN1 catalyses protein conformational changes that affect both APP and Tau processing [45]. Knockout of PIN1 increases amyloidogenic APP processing and intracellular A*β* levels in mice. PIN1-knockout mice also exhibit Tau hyperphosphorylation, motor and behavioural deficits, and neuronal degeneration [46].

Moreover, the cause of mitochondrial dysfunction could be the alteration of mitochondria-associated ER membrane (MAM) function. MAM is a dynamic subcompartment of ER with a lipid raft-like structure intimately involved in cholesterol and phospholipid metabolism, calcium homeostasis, mitochondrial function and dynamics, bioenergetics, and cell signaling [47–49]. MAM is physically, biochemically, and reversibly associated to mitochondria and this organellar contact site is a critical intracellular signaling platform that determines cellular life and death. During cellular stress situations, like an altered cellular redox state, the MAM alters its set of regulatory proteins and consequently alters MAM functions. Presenilins and *γ*-secretase are enriched in ER subcompartment and it has been hypothesized that genetic and biochemical alterations in these factors affecting MAM function should be relevant for increased APP processing and AD progression. Moreover, in agreement with this hypothesis both in model systems as PS-knockdown cells and in fibroblasts from patients having familial (FDA) or sporadic (SDA) forms of AD, MAM functionality, measured by the amount of cholesterol and phosphatidylserine production, is increased [50–52]. Similarly an increased expression of MAM-associated proteins is found in postmortem AD brains [53].

## 6. A**β** Affects Mitochondrial Bioenergetics 

The bioenergetics effect induced by endogenous A*β* on mitochondrion was investigated both in 7WD4 and 7PA2, two cell lines used as cellular model for AD [54]. Functional impairment of the respiratory chain was found distributed among the protein complexes especially in complex I and complex IV. Measurements of ATP concentration showed, in 7WD4 and 7PA2 cells, that its synthesis, by oxidative phosphorylation, was decreased by ~25%, and this loss was somewhat compensated by glycolysis (Warburg effect). Compensation proved to be more efficient in 7WD4 than in 7PA2 cells in agreement with the highest ROS production of the latter cell line. Moreover, the mitochondrial membrane potential was 40% and 60% lower in 7WD4 and 7PA2 cells, respectively, compared to Chinese hamster ovary (CHO) controls. Moreover, in different AD models, the voltage-dependent anion channel VDAC, a major component of the outer mitochondrial membrane that regulates ion fluxes and metabolites, is damaged as a result of oxidative stress [55]. The lipid composition of lipid rafts, key membrane microdomains that facilitate the transfer of substrates, and protein-protein and lipid-protein interactions is altered as a result of the abnormally low levels of n-3 long chain polyunsaturated fatty acids (mainly docosahexaenoic acid) that increase viscosity and augment energy consumption. Abnormal lipid raft composition may also modify the activity of the key enzymes that modulate the cleavage of the APP to form toxic A*β* [55]. Cholesterol and sphingolipids, that are rich in membrane microdomains, change their metabolism during normal brain aging and in the brains of AD patients resulting in accumulation of long chain ceramides and cholesterol [56]. Similarly, exposure of hippocampal neurons to A*β* induces membrane oxidative stress that perturbs cholesterol metabolism and activates sphingomyelinases, resulting in increased ceramide production. In contrast, treatment of neurons with *α*-tocopherol or an inhibitor of sphingomyelin synthesis prevents accumulation of ceramides and cholesterol and protects them against A*β* induced death [56].

All these alterations converge in a severe bioenergetics mitochondria impairment of the AD cells, with the extent of mitochondrial dysfunction being correlated with the accumulation of A*β* and oligomers.

Mitochondria are the principal site for the ATP production oxidative phosphorylation (OXPHOS) system. The mitochondrial OXPHOS machinery, whose components are produced by mitochondrial genome, is composed of five multisubunit complexes (complexes I–V). Several studies have shown that direct exposure to A*β* significantly impairs functionality of the mitochondrial electron transport chain (ETC). The ETC is essential to ATP production and its constituent enzyme complexes are a major source of ROS generation, especially when one or more of the enzyme complexes is inhibited [57–59]. Moreover, age-related bioenergetics deficit was described in female of 3xTg-AD mice aged from 3 to 12 months [60]. A decreased activity of OXPHOS, pyruvate dehydrogenase (PDH), and cytochrome *c* oxidase (COX) regulatory enzymes and increased oxidative stress and lipid peroxidation were found. Most of the effects on mitochondria appeared at the age of 9 months, whereas mitochondrial respiration was significantly decreased at 12 months of age. Importantly, mitochondrial bioenergetics deficit preexists at the development of AD pathology in 3xTg-AD mice.

APP is known to be alternatively spliced to produce three major isoforms in the brain: APP695, APP751, and APP770. Both APP770 and APP751 contain the Kunitz protease inhibitory (KPI) domain, but the former contains also an extra OX-2 domain. APP695 on the other hand lacks both domains. In AD, upregulation of the KPI-containing APP isoforms has been reported. Chua et al. found that the KPI-containing APP751 significantly decreased the expression of three major mitochondrial metabolic enzymes such as citrate synthase, succinate dehydrogenase, and cytochrome *c* oxidase (COX IV). This reduction lowers the NAD+/NADH ratio, COX IV activity, and mitochondrial membrane potential [61].

## 7. Mitochondrial Biogenesis and AD

During the life cycle, the biogenesis of new mitochondria plays an essential role in maintaining healthy mitochondria in eukaryotic cells. Mitochondrial biogenesis could also have the potentiality to quickly respond to changes due to mitochondrial damage or increased demand in response to environmental stimuli. Mitochondrial biogenesis is regulated by the PGC-1 *α*-NRFTFAM pathway. Expression levels of PGC-1 *α*, NRF 1, NRF 2, and TFAM were significantly decreased in both AD hippocampal tissue and APPswe M17 cells, suggesting that mitochondrial biogenesis was affected during neurodegeneration. Moreover, it has been demonstrated that impaired mitochondrial biogenesis contributes to mitochondrial dysfunction in AD and its enhancing may represent a potential pharmacologic approach for the treatment of AD [62].

## 8. Abnormal Mitochondrial Dynamics in AD

Despite traditional knowledge, mitochondria are now considered highly dynamic organelles that move throughout a cell and regularly fuse and divide. Increasing evidence suggests that abnormal mitochondrial dynamics, such as increased fission and decreased fusion, are early and key factors that have been found in neurodegenerative diseases, such as AD. These processes observed in axons and dendrites, as in all cells, allow the exchange of materials between mitochondria [63]. Even brief contact can involve fusion and extensive exchange of proteins in each compartment of the mitochondrion, as it has been shown in nonneuronal cells [64]. In addition, these abnormal mitochondrial dynamics have been associated with unusual changes in mitochondria structure. Abnormal mitochondrial fission and fusion were reported in AD [65]. In this study, the authors aimed to determine whether APP and A*β* cause mitochondrial and neuronal dysfunction through modulation of mitochondrial dynamics [65]. Further, both confocal and electron microscopy analyses demonstrated that APP overexpression causes mitochondrial fragmentation in neurons [66]. Since the balance of mitochondrial fission and fusion tightly controls mitochondrial morphology, it was hypothesized that APP-induced mitochondrial fragmentation was caused by enhanced fission and reduced fusion [67]. Recent studies show that the fusion dynamin-related protein 1 (Drp1), also known as dynamin-like protein 1 (DLP1), a protein that maintains and remodels mammalian mitochondria, interacts with A*β* and phosphorylates Tau, leading to excessive mitochondrial fragmentation, impaired axonal transport of mitochondria, and lastly neuronal damage and cognitive decline [68]. However, in agreement with the idea that mitochondria dynamics are altered in AD neurons, significant changes in the expression of proteins involved in mitochondrial fission and fusion were reported. Reduced expression of all the fusion proteins (i.e., OPA1, Mfn1, and Mfn2) and increased expression of fission protein Fis1 were described [69–71].

## 9. A**β** Interacts and Interferes with Mitochondrial Proteins 

Structurally and functionally intact mitochondria are crucial for healthy cells. A mitochondrion contains outer and inner membranes composed of phospholipid bilayers and proteins having different properties and their integrity is essential for its good functionality. In cell death mechanisms, as apoptosis and necrosis, an increase of mitochondrial membrane permeability is one of the key events. Mitochondria isolated from a variety of sources can show an abrupt increase in the permeability of the inner mitochondrial membrane to solutes with a molecular mass of less than 1,500 Da, which results in the loss of mitochondrial membrane potential (ΔΨm), mitochondrial swelling, and rupture of the outer mitochondrial membrane. This process is better known as the mitochondrial membrane permeability transition (MPT) [72]. The MPT can be induced under various conditions, such as exposure of mitochondria to Ca^2+^ together with inorganic phosphate. Although the molecular mechanisms of the MPT are largely unknown, the most widely accepted model is that it occurs after the opening of a channel complex that has been termed the permeability transition pore (PTP), which is thought to consist of the voltage-dependent anion channel (VDAC: outer membrane channel), the adenine nucleotide translocator (ANT: inner membrane channel), cyclophilin D (CypD), and possibly other molecule(s). CypD, a peptidylprolyl isomerase F, resides in the mitochondrial matrix but becomes associated with the inner mitochondrial membrane during the MPT and it plays a central role in opening the mitochondrial membrane permeability transition pore (mPTP). Recent studies provide evidence that CypD binds to A*β* and forms a complex with A*β* in cortical mitochondria of AD patients and Tg A*β*PP mice [73]. The interaction of A*β* with CypD provokes mitochondrial and neuronal perturbation [74]. This interaction causes the formation of mPTP, resulting in decreased mitochondrial membrane potential, compromised mitochondrial respiration function, increased oxidative stress, release of cytochrome *c*, and impaired axonal mitochondrial transport. It has been suggested that mPTP could be a possible therapeutic target. The design of small molecules able to interfere with A*β*-CypD complex could, indeed, decrease A*β* neurotoxicity effects [74].

The involvement of mitochondria in the pathogenic pathway of A*β* was also confirmed by specific binding of A*β* and A*β*PP to mitochondrial proteins, which causes energy impairment and cell physiology defects. Firstly, A*β* specifically binds to the mitochondrial A*β*-binding alcohol dehydrogenase (ABAD), an intracellular enzyme present in the mitochondrial matrix [75]. In presence of A*β*, ABAD increases cell stress, DNA fragmentation, and ROS generation induced by A*β*. Moreover, the expression levels of ABAD are related to mitochondrial A*β* levels being ABAD expression levels significantly higher in AD-affected brain regions, as hippocampus, than in healthy brain [75]. Recent experiments indicate that the 94–114 ABAD residues interact with A*β* and inhibition of the ABAD-A*β* interaction significantly reduces mitochondrial A*β* accumulation and protects against aberrant mitochondrial and neuronal function, improving spatial learning/memory in Tg AD mice [76–78]. Proteomics studies on Tg mAPP/ABAD mice showed that in brain the ABAD-A*β* interaction also affects the expression of proteins such as Ep-1 (endophilin-1), a cytoplasmic SH3 domain-containing protein, located in presynaptic nerve termini [79] and Prdx-2 (peroxiredoxin-2), an antioxidant protein [80], both of which were also found to be overexpressed in human AD brains [79, 80]. Even if the link between these two proteins and mitochondrial dysfunction remains unclear some evidences indicate that increased levels of Ep-1 cause the activation of JNK (c-Jun-N-terminal kinase) [79, 81]. JNK is a stress kinase that has been linked to A*β* production and neurons death [82]. Thus, all these data support the significance of ABAD to A*β* induced neuronal stress. Several enzymes, such as presequence protease (PreP), a metalloprotease containing an inverted zinc binding motif, were identified to degrade A*β* [83, 84]. Intramitochondrial localization studies demonstrated that PreP is localized within the mitochondrial matrix. PreP activity is severely reduced in samples of mitochondrial matrix isolated from the temporal lobe of AD patients, compared to age-matched controls [85]. Moreover, exposure of purified hPreP to an oxidizing agent, hydrogen peroxide, results in decreased peptidolytic activity [86]. During oxidative phosphorylation, the electron transporters release energy that drives the production of ATP. A*β* may inhibit mitochondrial respiration, by interacting with the subunit *α* of ATP synthase causing ATP depletion [87]. Another study reports that A*β* decreases cytochrome *c* oxidase activity, but the specific mechanism is still unclear [88]. In contrast, an increase in the activity of the cytochrome *c* reductase (complex III) has been reported [89]. Interestingly, complex III appears to be one of the sites of superoxide radical production. Bobba et al. found that A*β* caused a selective defect in complex I activity, associated with an increase of intracellular ROS (5-fold) and an impairment of complex IV, likely due to membrane lipid peroxidation. In addition, a 130% increase of the GSSG/GSH ratio was measured in AD brains with respect to age-matched controls [90].

## 10. Therapeutic Strategies against A*β* Induced Mitochondrial Toxicity 

The free radical and oxidative stress theory of aging suggests that oxidative damage is a major player in neuronal degeneration and oxidative stress has a well-established pathophysiological feature in AD [91]. However, up to now use of antioxidants in prevention or therapy gives controversial results. One possible explanation is related to the low permeability of the blood brain barrier (BBB) to most of the currently used antioxidants. To overcome these potential difficulties, researchers have formulated new delivery systems, such as those based on nanoparticles, which might represent a successful strategy for drug delivery into CNS. Moreover, on the basis of the consideration that mitochondria are the major source of ROS and are particularly vulnerable to oxidative stress, one would predict that a more efficient therapy could consider use of antioxidants alleviating mitochondrial dysfunction. This prompted researchers to develop antioxidant therapy directed to mitochondrion, through the development of specifically designed mitochondria-targeted antioxidants [92, 93]. This might be a strategy to overcome the apparent clinical inefficiency of antioxidants that do not target oxidative stress in this organelle [94]. The Szeto-Schiller (SS) peptides, a family of small mitochondria-targeted antioxidant molecules, were developed as a potential treatment for AD [95]. These SS peptides display mitochondrial accumulation having a sequence motif that allows them to target mitochondria. They scavenge H_2_O_2_ and inhibit lipid peroxidation. Their antioxidant action can be attributed to the tyrosine, or dimethyltyrosine (Dmt), that plays a role in scavenging mitochondrial ROS. SS31, in particular, is capable of entering mitochondria and of concentrating in the inner mitochondrial membrane, protecting mitochondria against mPTP formation, swelling, and cytochrome *c* release. Using SS31 in different AD models as N2a neuroblastoma cells treated with A*β*_25–35_, primary neurons from Tg2576 mice and aged Tg2576 mice, a range of effects on mitochondria were observed [96, 97]. Increased expression of mitochondrial fission genes and decreased expression of fusion genes were found together with increased number of mitochondria, indicating that mitochondria fragmentation occurred. On the basis of these results, the Antipodean Pharmaceuticals Inc. patented MitoQ, a mitochondria-targeted antioxidant. This drug is now undergoing phase II clinical trials for the potential treatment of several diseases in which mitochondrial oxidative damage is implicated, including neurodegenerative diseases [98]. MitoQ was designed to accumulate extensively within mitochondria* in vivo* in order to increase the local antioxidant capacity against oxidative damage. The active drug is the ubiquinone, which is identical to the antioxidant component of the respiratory chain constituent coenzyme Q10 (CoQ10). An aliphatic 10-carbon chain, to the lipophilic cation triphenylphosphonium, that drives its selective uptake into mitochondria in a membrane potential-dependent manner, covalently links this component. Once internalized by mitochondria, it adsorbs in the phospholipid bilayers, where it is readily reduced to the active ubiquinol form MitoQH2, which exerts its antioxidant properties [99]. Since it enters into mitochondria several hundred-fold more than natural antioxidants, it rapidly neutralizes free radicals at their source, before they reach their targets, thus showing an improved therapeutic potential [100]. For all these proprieties, MitoQ is a promising antioxidant candidate for AD treatment [94]. Recently, using cytoplasmic hybrid (cybrid) neurons from AD and age-matched non-AD human subjects, it was demonstrated that treatment with the antioxidant probucol protects against AD mitochondria-induced extracellular signal-regulated kinase (ERK) activation and mitochondrial fission-fusion imbalances. In fact, inhibition of ERK activation not only attenuates aberrant mitochondrial morphology and function but also reestablishes the mitochondrial fission and fusion balance, as confirmed by changes in expression and distribution of DLP1 and Mfn2 [101].

Among antioxidant molecules used in prevention and treatment of AD, a relevant role is played by natural antioxidants [102]. The curry spice curcumin shows antioxidant [103], anti-inflammatory [104], and amyloid-disaggregating properties [105], effects that have been largely studied both* in vitro* and* in vivo* models. A pilot clinical trial to develop procedures for testing the effectiveness of curcumin on slowing AD progression was carried out [106]. Thirty-four human subjects with AD received daily placebo or two different doses of curcumin for 6 months. At different times of the study, cognitive tests were performed, and blood samples were analyzed for levels of isoprostane, amyloid beta protein, metals, and cholesterol. There was no cognitive decline in the placebo group, and no improvement was observed with curcumin. Moreover, curcumin presented low bioavailability and photodegradation. With such issues in mind, Marrache and Dhar formulated targeted curcumin-loaded NPs (mitochondria-targeted polymeric nanoparticle system) to provide photostability and enhance mitochondrial uptake. An* in vitro* cytotoxicity assay, using A*β* treated human neuroblastoma IMR-32 cells, demonstrated enhanced neuroprotection with the targeted curcumin NPs compared with the no-targeted curcumin NPs or free curcumin against A*β*, which accounts for the targeted delivery of curcumin into the mitochondria of cells [107]. Ferulic acid (FA) is another natural antioxidant having a neuroprotective effect against oxidative stress and cell death induced by A*β*_42_ oligomers [36]. FA was also successfully conjugated with solid lipid nanoparticles to improve its delivery and enhance its potential antioxidant therapeutic effect [36]. Moreover, other data suggest that natural plants such as a standardized* Ginkgo biloba* extract or the green tea component epigallocatechin-3-gallate may be promising treatment strategies. Of note, in addition to their antioxidative properties, these compounds stabilize mitochondrial functions such as the mitochondrial membrane potential, ATP levels, and mitochondrial respiratory complexes [108, 109]. As discussed above, the interaction of A*β* with CypD provokes mitochondrial perturbation and formation of mPTP events leading to neuronal degeneration. Thus, inhibition of mPTP formation by blocking CypD is a rational target for potential therapeutic AD strategies.

## 11. Conclusions

**Figure 2:**
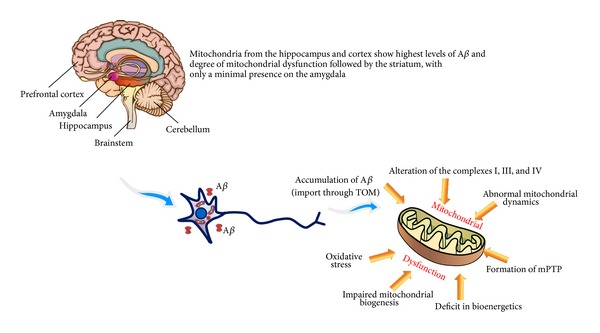
Mitochondrial dysfunction induced by A*β*. In AD brain some areas are more sensitive to mitochondrial dysfunction. In the neurons A*β* induces mitochondrial dysfunction by using different mechanisms. A*β* is taken up by mitochondria via the TOM complex and imported in the inner membrane; A*β* alters the enzyme activity of the respiratory chain complexes I, II, and IV; A*β* affects mitochondrial dynamics by impaired balance of fission and fusion; A*β* causes formation of mPTP via interaction with CypD; A*β* induces decreased mitochondrial respiration; A*β* affects new mitochondrial biogenesis; A*β* increases ROS generation.

Mitochondrial dysfunction is an early feature of Alzheimer's disease. Extracellular or intracellular A*β* is imported into the mitochondria through the TOM machinery. The progressive accumulation of mitochondrial A*β* is associated with aberrant mitochondrial functions leading to neuronal damage and cognitive decline. The mitotoxicity induced by A*β* is still not clear but includes numerous mechanisms. A*β* induced mitochondrial dysfunction contributes to energy metabolism impairment, defects in key respiratory enzyme activity/function, accumulation/generation of mitochondrial ROS, formation of mPTP, altered mitochondrial biogenesis, and dynamics. Binding of A*β* to mitochondrial proteins (CypD and ABAD) amplifies A*β* induced effects on mitochondria and neuron functions (Figure 2). From these insights, it is easy to deduce how mitochondrion offers multiple points to develop strategies against mitochondrial dysfunction. Thus, not only antioxidant targeted therapeutic strategies but also specific mitochondrial targeted therapeutic strategies should be explored for neuroprotection against A*β* toxicity.
